# Pharmacokinetic/Pharmacodynamic Integration to Evaluate the Changes in Susceptibility of *Actinobacillus pleuropneumoniae* After Repeated Administration of Danofloxacin

**DOI:** 10.3389/fmicb.2018.02445

**Published:** 2018-10-10

**Authors:** Longfei Zhang, Zheng Kang, Lihua Yao, Xiaoyan Gu, Zilong Huang, Qinren Cai, Xiangguang Shen, Huanzhong Ding

**Affiliations:** ^1^Guangdong Key Laboratory for Veterinary Drug Development and Safety Evaluation, South China Agricultural University, Guangzhou, China; ^2^Technical Center for Inspection and Quarantine, Zhuhai Entry-Exit Inspection and Quarantine Bureau, Zhuhai, China

**Keywords:** PK/PD, mutant frequency, danofloxacin, *Actinobacillus pleuropneumoniae*, tissue cage infection model

## Abstract

To evaluate the relationship between pharmacokinetic/pharmacodynamic (PK/PD) parameters and changes in susceptibility and resistance frequency of *Actinobacillus pleuropneumoniae* CVCC 259, a piglet tissue cage (TC) infection model was established. After *A. pleuropneumoniae* populations maintained at 10^8^ CFU/mL in TCs, piglets were treated with various doses of danofloxacin once daily for 5 consecutive days by intramuscular injection. Both the concentrations of danofloxacin and the population of vial cells were determined. Changes in susceptibility and resistance frequency were monitored. Polymerase chain reaction (PCR) amplification of quinolone resistance-determining regions (QRDRs) and DNA sequencing were performed to identify point mutations in *gyrA*, *gyrB*, *parC*, and *parE* genes. Furthermore, the susceptibility of mutants to danofloxacin and enrofloxacin was determined in the presence or absence of reserpine to assess whether the mutants were caused by efflux pumps. The MICs and resistant frequency of *A. pleuropneumoniae* both increased when danofloxacin concentrations fluctuated between MIC_99_ (0.05 μg/mL) and MPC (mutant prevention concentration, 0.4 μg/mL). As for PK/PD parameters, the resistant mutants were selected and enriched when AUC_24h_/MIC_99_ ranged from 34.68 to 148.65 h or AUC_24h_/MPC ranged from 4.33 to 18.58 h. Substitutions of Ser-83→Tyr or Ser-83→Phe in *gyrA* and Lys-53→Glu in *parC* were observed. The susceptibility of mutants obtained via danofloxacin treatment at 1.25 and 2.5 mg/kg were less affected by reserpine. These results demonstrate that maintaining the value of AUC_24h_/MPC above 18.58 h may produce a desirable antibacterial effect and protect against *A. pleuropneumoniae* resistance to danofloxacin.

## Introduction

*Actinobacillus pleuropneumoniae* is the causative agent of porcine pleuropneumonia, a severe respiratory disease that is a global problem in pig production. The acute form of this disease is highly contagious and often fatal, resulting in considerable economic losses to pig producers (Gutiérrez-Martín et al., 2006; Matter et al., 2007; Bossé et al., 2015). Historically, antibacterial therapy was a highly effective and common measure in controlling this disease. However, resistant mutants increased gradually due to the misuse of antibacterials. According to a recent report, the MIC frequency distribution of danofloxacin against *A. pleuropneumoniae* gradually increased during 2011–2015 in both the United States and Canada (Sweeney et al., 2017). Therefore, a rational antibiotic dosing regimen should be optimized, not only to eradicate bacterial infections but also to inhibit the emergence and proliferation of antibiotic-resistant strains (Toutain et al., 2002).

To design more rational dosage schedules, the antibacterial effect and pharmacokinetics of antibiotic should be considered integratedly (Aliabadi and Lees, 2000; Lees and Aliabadi, 2002). Therefore, the pharmacokinetic/pharmacodynamic (PK/PD) integration model has been commonly used as an alternative and preferred approach to dose titration studies for selection of rational dosage regimens (Toutain and Lees, 2004). To restrict selection of antibiotic-resistant mutants, various methods have been proposed. For PK/PD integration, the MIC- and MPC-related PK/PD parameters (MPC:MIC for the least susceptible single-step mutant subpopulation) have an important role in understanding the development of resistance (Firsov et al., 2003). Indeed, the relationship between PK/PD parameters and resistant mutants has been studied in several *in vitro* experiments (Firsov et al., 2003; Zinner et al., 2003, 2008; Liang et al., 2011). For *in vivo* experiments, a tissue cage (TC) infection model has been used as a feasible system in exploring the relationship between PK/PD parameters and antibacterial effects (Cui et al., 2006; Zhu et al., 2012; Zhang et al., 2014a; Xiong et al., 2016).

Danofloxacin is a third-generation quinolone with a broad-spectrum bactericidal activity and used solely in veterinary. The pharmacokinetics of danofloxacin has been investigated in several animals, such as sheep (Aliabadi et al., 2003b), goats (Aliabadi and Lees, 2001), calf (Sarasola et al., 2002), camel (Aliabadi et al., 2003a), and pigs (Richez et al., 1997). To design rational dosage regimen, the PK/PD integration model of danofloxacin against pathogenic microorgnism has been studied. A TC model was well applied to explore the antibacterial activity of danoflocaxin against bacteria, especially in ruminant. For example, one group (Aliabadi et al., 2003b) has studied the antibacterial activity of danoflocaxin against *Mannheimia haemolytica* in sheep biological fluids. After integrating the antibacterial effect and PK/PD parameters, the mean values of AUC/MIC to produce bacteriostasis, bactericidal activity, and elimination of bacteria were 17.8, 20.2, and 28.7 h for serum and 20.6, 25.5, and 41.6 h for exudate, respectively. Another study (Shojaee and Lees, 2003) focused on the PK/PD integration of danofloxacin against *M. haemolytica* 3575 in calf and the mean values of AUC/MIC to produce a bacteriostatic effect, inhibition of bacterial count by 50%, bactericidal effect, and elimination of bacteria were 15.9, 16.7, 18.15, and 33.5 h for serum and 15.0, 16.34, 17.8, and 30.7 h for transudate, respectively. In camel (Aliabadi et al., 2003a), the PK/PD modeling of danofloxacin against *Escherichia coli* 0157-H7 was developed in serum and TC fluids and the mean values of AUC_-_/MIC to produce a bacteriostatic activity, inhibition of bacterial count by 50%, bactericidal activity, and elimination of bacteria for serum were 17.20, 20.07, 21.24, and 68.37 h, respectively. A goat TC model (Aliabadi and Lees, 2001) has been used to estimate the antibacterial activity of danofloxacin against *M. haemolytica* and the mean values of AUC_24_/MIC in serum to produce bacteriostasis, bactericidal effect, and elimination of bacteria were 22.6, 29.6, and 52.2 h, respectively. These studies provided abundant and original PK/PD data, which are of great significance for guiding the clinical medication of danofloxacin in animals. However, there is no paper about PK/PD integration of danofloxacin in pigs and there is also no report about correlation analysis between PK/PD parameters of danofloxacin and bacterial sensitivity changes. Therefore, PK/PD integration was developed to evaluate the changes in susceptibility of *A. pleuropneumoniae* after repeated administration of danofloxacin in pigs in this manuscript.

In the present study, a standard *A. pleuropneumoniae* CVCC 259 strain was exposed to various doses of danofloxacin in a piglet TC infection model at a population of 10^8^ CFU/mL. The pharmacokinetics of danofloxacin and the changes in susceptibility and resistance frequency of *A. pleuropneumoniae* were examined. We then identified the mutations in the quinolone resistance-determining regions (QRDRs) of *gyrA*, *gyrB*, *parC*, and *parE* genes. Finally, the relationship between PK/PD parameters and changes in susceptibility and resistance frequency of *A. pleuropneumoniae* was analyzed. We aimed to demonstrate that this model could elucidate the relationship between emergence of resistant *A. pleuropneumoniae* and PK/PD parameters associated with danofloxacin.

## Materials and Methods

### Bacterial Strain, Antibacterial Agents, and Chemicals

The *A. pleuropneumoniae* standard strain, CVCC259, was purchased from the Chinese Veterinary Culture Collection Center. Danofloxacin mesylate standard (>99%) and enrofloxacin standard (98%) were kindly supplied by Guangdong Dahuanong Animal Health Products. Pentobarbital sodium was purchased from Jian Yang Biotechnology Co., Ltd. Procainamide hydrochloride was supplied by Xin Zheng Co., Ltd., Tianjin Pharmaceutical Group. Tryptic Soy Broth (TSB) and Mueller–Hinton agar (MHA) were purchased from Guangdong Huankai Microbial Technology. Nicotinamide adenine dinucleotide (NAD, lot: 20160810) was purchased from MYM biological technology company limited (Beijing). Newborn bovine serum was provided by Guangzhou Ruite Biotechnology Ltd. Compound aminopyrine injection was purchased from Shandong Zhengmu Biological Pharmaceutical Co., Ltd.

### Determination of MIC, MIC_99_, and MPC

*Actinobacillus pleuropneumoniae* was grown in TSB or on MHA supplemented with 4% newborn bovine serum and 1% NAD at 1 mg/mL. The MIC was tested by an agar dilution method according to Clinical and Laboratory Standards Institute (CLSI) reference methods (Watts, 2013). MIC_99_ and MPC were determined as previously described (Lu et al., 2003) with minor revision. Briefly, for MIC_99_, bacterial cultures were grown for 8 h at a constant temperature of 37°C, at 180–200 rpm/min. Cultures were serially diluted and a 100 μL inoculum with a concentration of bacteria at approximately 10^6^ CFU/mL was applied to agar plates containing various concentrations of danofloxacin. After incubation at 37°C, 5% CO_2_ for 18–20 h, bacterial colonies were counted, and the fraction relative to the initial bacterial inoculum was calculated. The MIC is recorded as the lowest drug concentration preventing visible growth. The MIC_99_ is defined as the drug concentrations that inhibited growth of bacteria by 99%.

For MPC, approximately 10^10^ CFU *A. pleuropneumoniae* were inoculated on to multiple danofloxacin-containing agar plates. After incubation at 37°C for 72 h, plates were screened every 24 h. The lowest antibiotic concentration at which no colonies grew on an agar plate was defined as the preliminary MPC (MPC_pr_). For exact MPC, the concentrations of danofloxacin in the agar decreased at a linear trajectory by 10%, which was based on MPC_pr_ approaching 1/2 MPC_pr_. Then, we repeated the method for the MPC_pr_ test. The lowest antibiotic concentration at which no colonies grew on an agar plate was defined as the MPC.

### Tissue Cage Infection Model

Healthy castrated crossbred piglets (Duroc × Landrace× Yorkshire), weighing 20–25 kg, were obtained from Guangzhou Fine Breed Swine Farm. They were housed in individual cages and fed antibiotic-free fodder (guangchubao premix feed for pig from the Guangzhou Zhongwang Feed Company) twice a day. Water was available *ad libitum*. All the experimental protocols were approved by the South China Agricultural University Committee on Animal Ethics (Approval number: 2017A008).

Tissue cages were made using food grade silicone tubes and the size of the TCs were the same as those described previously (Zhang et al., 2014b). Implantation surgery was performed under deep general anesthesia induced by pentobarbital sodium and local anesthesia by the injection of procainamide hydrochloride. Two TCs, sterilized with 75% ethyl alcohol and ultraviolet radiation, were implanted subcutaneously in each piglet. The TC position was perpendicular to the horizontal plane and one TC was placed on each side of the neck equidistant from the jugular vein and spinal cord. After surgery, the piglets received intramuscular (IM) injection of penicillin (1,000,000 IU/kg) to prevent infection. Animals were also treated with tetracycline ointment over the wound twice a day for 3 days. The non-steroidal anti-inflammatory drug (NSAID), aminopyrine, was simultaneously administrated by injection for post-operative analgesia. The animals were allowed to recover from surgery for 4–5 weeks to permit wound recovery and for the TC to fill with tissue cage fluid (TCF). After extraction of the TCF with disposable sterile syringes and bacteriological examination, sterile TCs were used for the study.

One milliliter of logarithmic growth phase bacterial suspension (approximately 10^10^ CFU/mL) was added to each TC. Two days after infection, 0.5 mL of TCF was extracted from each TC for bacterial enumeration. The TCFs containing a bacterial concentration exceeding 10^8^ CFU/mL were used for the experiment.

### Dosing Regimens and Pharmacokinetic Measurements

Sixteen piglets (eight females and eight males) were randomly allocated to one control group and seven study groups. The control group (two piglets and four TCs) was treated with 1 mL sterile physiological saline. The study groups were treated with danofloxacin at 0.4, 0.6, 0.8, 1.25, 2.5, 3.5, and 5 mg/kg (four TCs for each group) of body weight for 5 days, once daily by IM injection. TCFs (0.3 mL) were collected from the TC at 2, 4, 6, 8, 10, and 24 h after each administration. Samples were clarified by centrifugation at 3000 × *g* for 10 min and stored at -20°C avoiding light until analyzed within 2 weeks.

Danofloxacin concentrations in TCF were determined by high-performance liquid chromatography with fluorescence detection (HPLC-FD; Agilent Technologies, United States; Zhang et al., 2017). Briefly, after thawing, each sample (200 μL) including the blank sample was added to the same volume of acetonitrile for deproteinization, and was then clarified by centrifugation at 12,000 ×*g* for 10 min. Two-hundred microliters of clear supernatant and 800 μL water were mixed and then transferred to an HPLC vial. The HPLC was applied with an Agilent TC-C18 column (250 mm × 4.6 mm, 5 μm) and the mobile phase was triethylamine phosphate (pH 2.4): acetonitrile (19:81, v/v) with a flow rate of 0.8 mL/min. The injection volume was 20 μL. A calibration curve was determined using nine danofloxacin concentrations (0.001–0.5 μg/mL). The mean relative recovery (RR) of danofloxacin in TCF samples was 96.9 ± 9.83% (mean ± SD).

Pharmacokinetic parameters, including C_max_ (maximum concentration of drug in samples) and AUC_24h_ (the area under the concentration–time curve over 24 h), were calculated by the non-compartmental model using WinNonlin software (version 5.2, Pharsight Corporation, Mountain View, CA, United States).

### Quantification of the Time-Kill Curves and Recovery Curves of Resistant Mutants

Multiple TCFs (0.5 mL) were collected from the TCs before, during, and after the treatment (after every administration) at 24 and 48 h after the termination of treatment. To quantify the numbers of surviving bacteria and resistant mutants, each sample was serially diluted with sterile saline and 20 μL was inoculated in triplicate on to drug-free MHA or MHA containing 1 × MIC of danofloxacin. After incubation 18–20 h, the resultant bacterial colonies were counted. The detection limit was 50 CFU/mL. The time-kill curves were depicted as the number of bacteria on drug-free MHA, while the recovery curves of resistant mutants were drawn as the populations grown on MHA containing 1 × MIC of danofloxacin.

### Quantification of Changes in Susceptibility and Resistant Frequency

Loss of bacterial susceptibility in TCF was examined at before danofloxacin administration, during the treatment (after every administration), 24 and 48 h after the termination of treatment. The stability of mutants was determined by consecutive passage of *A. pleuropneumoniae* on to drug-free MHA every 24 h for 5 days. MICs were tested as described above. To evaluate the contribution of efflux, the susceptibility to both danofloxacin and enrofloxacin was then determined in the presence or absence of reserpine at 20 μg/mL.

To detect the resistant frequency of mutants, each sample was plated on to MHA containing 1 × MIC of danofloxacin (detection limit 50 CFU/mL). The definition of resistant frequency was expressed by the ratio of bacterial numbers counted in the presence of antibiotics to that in the absence of antibiotics.

### Analysis of the Relationship Between PK/PD Parameters and Resistant Mutants

Pharmacokinetic/pharmacodynamic parameters such as AUC_24h_/MIC_99_, AUC_24h_/MPC, %T > MIC_99_ (the percentage of the time that drug concentration remains above the MIC_99_), %T > MPC (the percentage of time that drug concentration remains above the MPC), C_max_/MIC_99_, and C_max_/MPC were calculated using WinNonlin program (version 5.2, Pharsight Corporation, Mountain View, CA, United States). Fisher’s exact test was used for statistical analysis of the relationship between PK/PD indices and the changes in susceptible. Control group (two piglets and four TCs) were used as a control. *P* < 0.05 was considered to be statistically significant.

### PCR Amplification of Quinolone Resistance-Determining Regions (QRDRs)

After passage for five generations, mutants with stable MIC were used for polymerase chain reaction (PCR) amplification. The nucleotide sequence of the QRDRs of the *gyrA*, *gyrB*, *parC*, and *parE* genes were determined as previously described (Wang et al., 2010). The reagents used for PCR were purchased from Takara Bio, (Kusatsu, Japan). After amplification, the sequencing reaction was analyzed by Beijing Genomics Institute using Sanger sequencing.

## Results

### MIC, MIC_99_, and MPC of Danofloxacin Against *A. pleuropneumoniae*

The values of MIC, MIC_99_, and MPC were 0.06, 0.05, and 0.4 μg/mL, respectively. All experiments were performed in triplicate on different occasions.

### Antibacterial Effect and Recovery of Resistant Mutants

**FIGURE 1:**
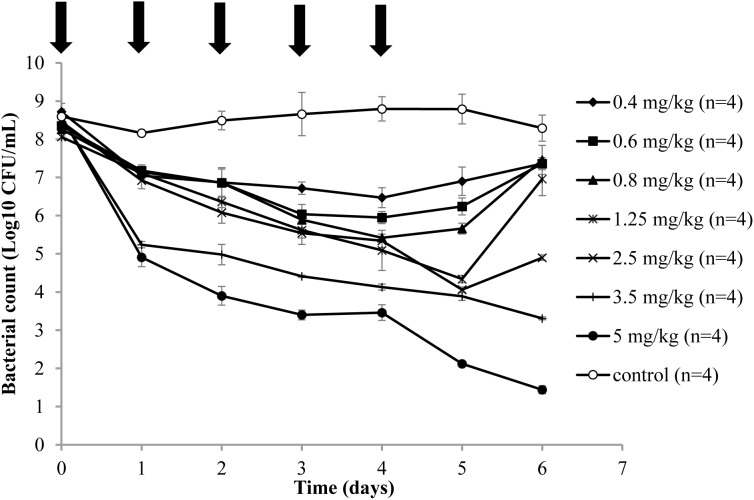
Time-killing curves in the tissue cage fluid of danofloxacin treatment of *A. pleuropneumoniae* CVCC259. After an infection model was established, various doses (0.4, 0.6, 0.8, 1.25, 2.5, 3.5, and 5 mg/kg of body weight by IM) of danofloxacin and sterile physiological saline (control group) were administered intramuscularly once daily for 5 days (indicated by the arrow). *n* is the number of tissue cages per group.

The time-kill curves are depicted in **Figure 1** and exhibit the antibacterial effect of danofloxcin against *A. pleuropneumoniae* CVCC259 in TCF after different doses were administered. For the control group, bacterial populations remained constant (approximately 10^8^ CFU/mL). Compared to the control group, administration of danofloxacin at 0.4 mg/kg slightly decreased bacterial numbers. Bacterial numbers were reduced in response to the first 4 administrations of danofloxacin at 0.4, 0.6, and 0.8 mg/kg, although there was re-growth of bacteria after the last treatment. For the danofloxacin dosages at 1.25 and 2.5 mg/kg, bacterial numbers were obviously reduced after the five administrations, although there was re-growth at 48 h after the last administration. Administration of danofloxacin at 3.5 and 5 mg/kg caused bacterial numbers to reduce throughout treatment and they remained low during the growth recovery phase.

**FIGURE 2:**
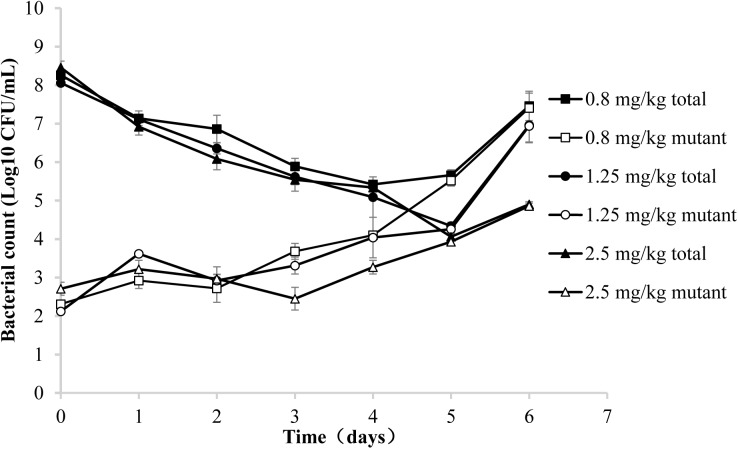
Recovery of total and resistant bacteria after administration of danofloxacin at 0.8, 1.25, and 2.5 mg/kg. Concentrations of total bacteria and resistant mutants were determined in aliquots of tissue cage fluid obtained at the indicated time points after the initiation of treatment. Three representative examples are shown for piglets in which the danofloxacin concentrations were located between the MIC_99_ and MPC.

Three representative recovery curves are shown in **Figure 2** when the piglets were administrated danofloxacin at 0.8, 1.25, and 2.5 mg/mL. As a result, the danofloxacin concentrations were located between the MIC_99_ and MPC. Both the numbers of total and resistant bacteria are listed in **Figure 2**. The total bacterial populations reduced during treatment and then gradually increased. However, resistant bacteria numbers were initially constant or slightly reduced before amplification after several administrations. At last, the number of mutant and total bacteria were almost equal.

### Pharmacokinetics of Danofloxacin

**FIGURE 3:**
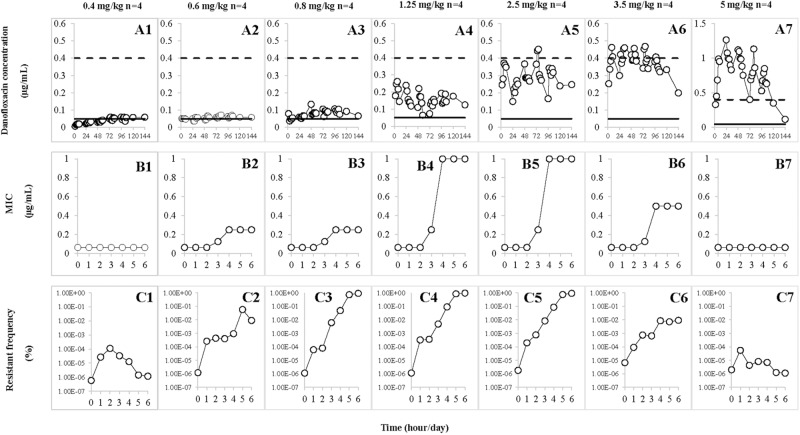
Effect of danofloxacin concentration on loss of susceptibility and mutant enrichment. Concentrations of danofloxacin (0.4, 0.6, 0.8, 1.25, 2.5, 3.5, and 5 mg/kg) correspond to the different treatment groups once daily for 5 days. *n* is the number of tissue cages per group. **(A1–A7)** The concentration–time curves of different dosages of danofloxacin. **(B1–B7)** The values of MIC after each administration of danofloxacin, which corresponded to **A1–A7**. **(C1–C7)** The resistant frequency after each administration of danofloxacin, which corresponded to **A1–A7**. The dotted line represents MPC and the solid line represents MIC_99_ in **A1–A7**.

Danofloxacin concentrations collected at various time points during the treatment are depicted in **Figures 3A1–A7**. Determined by trapezoidal rules, the average values of AUC_24h_ ranged from 0.96 ± 0.34 to 18.94 ± 3.34 μg⋅h/mL. The average maximum concentration (C_max_) ranged from 0.05 ± 0.01 to 1.13 ± 0.15 μg/mL. The detailed values for AUC_24h_ and C_max_ are listed in **Table 1**. The AUC_24h_ and C_max_ values in the TCF increased in a non-linear fashion with increasing doses and the correlation coefficients (*R*^2^) were 0.95 and 0.91, respectively. After various dosages of danofloxacin were administered, the mean concentrations in the TCFs were ranged from MIC_99_ to MPC: almost completely below MIC_99_ (A1), across the MIC_99_ (A2), completely between MIC_99_ and MPC (A3–A5), across the MPC (A6), and above the MPC (A7).

**Table 1:** The pharmacokinetic parameters of danofloxacin following multiple doses in a piglet tissue-cage infection model.

Dosages (mg/kg)	AUC_24h_ (μg⋅h/mL)	C_max_ (μg/mL)
0.4	0.96 ± 0.34	0.05 ± 0.01
0.6	1.35 ± 0.15	0.06 ± 0.01
0.8	1.98 ± 0.35	0.11 ± 0.02
1.25	3.59 ± 0.69	0.22 ± 0.03
2.5	6.79 ± 0.35	0.38 ± 0.04
3.5	9.00 ± 0.73	0.45 ± 0.02
5	18.94 ± 3.34	1.13 ± 0.15


*AUC_24h_, 24 h area under concentration–time curve; C_*max*_, maximum concentration. The AUC_*24h*_ and C_*max*_ were the mean values of five injections of danofloxacin at various dosages. Values are listed as mean ± SD.*

### Changes in Susceptibility and Resistant Frequency

Susceptibility of *A. pleuropneumoniae* in the TCFs was examined after administration with different doses of danofloxacin (**Figures 3B1–B7**). The MICs gradually increased (**Figures 3B2–B6**) when the drug concentrations were partially or completely located between MIC_99_ and MPC (**Figures 3A2–A6**). The significant increase in MICs (**Figures 3B4,B5**) were observed when the concentration of danofloxacin fluctuated between the MIC_99_ and MPC (**Figures 3A4,A5**). When danofloxacin concentrations were maintained either below the MIC_99_ (**Figure 3A1**) or above the MPC (**Figure 3A7**), MIC did not increase, either during or after treatment (**Figures 3B1,B7**).

The resistant frequencies are depicted in **Figures 3C1–C7**. Dramatic increases (>1000 fold; **Figures 3C2–C6**) were observed when the drug concentration located between MIC_99_ and MPC (**Figures 3A2–A6**). When drug concentrations were mostly below the MIC_99_ (**Figure 3A1**) or exceeded the MPC (**Figure 3A7**), the resistant frequencies slightly increased and then gradually decreased (**Figures 3C1,C7**).

### Relationships Between PK/PD Parameters and Resistant Mutants

Pharmacokinetic/pharmacodynamic parameters provide an empirical way to relate antimicrobial dose to favorable treatment effects associated with bactericidal agents (Mouton et al., 2005). The MIC_99_- and MPC-related PK/PD parameters are listed in **Table 2**. Relationships between PK/PD indices, determined as steady-state values after the fifth dose, and changes in susceptibility are shown in **Table 3**. For fluoroquinolones, the AUC_24h_/MIC_99_ index is most commonly associated with restriction of susceptible bacterial growth (Craig, 2001). Only two of eight TCs lost susceptibility when AUC_24h_/MIC_99_ < 34.68 h (**Table 3** and **Figures 3A1,A2**). Loss of bacterial susceptibility occurred in 10 of 12 TCs when AUC_24h_/MIC_99_ was between 34.68 and 148.65 h (**Table 3** and **Figures 3A3–A5**). Only one of eight TCs lost susceptibility when the AUC_24h_/MIC_99_ exceeded 148.65 h (**Table 3** and **Figures 3A6,A7**). As for AUC_24h_/MPC, mutant enrichment was observed, where 10 of 12 TCs lost susceptibility, when the AUC_24h_/MPC was between 4.33 and 18.58 h (**Table 3** and **Figures 3A3–A5**). Only one of eight TCs occurred loss of susceptibility (**Table 3** and **Figures 3A6,A7**) when AUC_24h_/MPC > 18.58 h.

**Table 2:** The PK/PD parameters of danofloxacin following multiple doses in a piglet tissue-cage infection model.

Dosages(mg/kg)	AUC_24h_/MIC_99_(h)	AUC_24h_/MPC(h)	C_max_/MIC_99_	C_max_/MPC	%T > MIC_99_	%T > MPC
0.4	19.18 ± 6.73	0.91 ± 0.30	2.40 ± 0.84	0.11 ± 0.04	1.74 ± 3.77	0
0.6	26.95 ± 3.09	1.28 ± 0.15	3.37 ± 0.39	0.16 ± 0.02	72.23 ± 8.32	0
0.8	39.52 ± 7.02	2.25 ± 0.41	4.94 ± 0.88	0.28 ± 0.05	94.42 ± 11.16	0
1.25	71.79 ± 13.72	4.44 ± 0.53	8.97 ± 1.72	0.55 ± 0.07	100	0
2.5	135.87 ± 6.91	7.60 ± 0.75	16.98 ± 0.86	0.95 ± 0.09	100	15.96 ± 6.38
3.5	179.95 ± 14.53	9.02 ± 0.44	22.49 ± 1.82	1.13 ± 0.05	100	29.63 ± 25.61
5	378.81 ± 66.86	22.55 ± 3.01	47.35 ± 8.36	2.82 ± 0.38	100	98.01 ± 3.98


*AUC_24h_, 24-h area under concentration–time curve; C_*max*_, maximum concentration; MIC_*99*_, the minimum concentration that inhibits colony formation by 99%; MPC, antibacterial concentration that inhibits growth of the least susceptible single-step mutant subpopulation; %T > MIC_*99*_, the percentage of time that drug concentration remained above MIC_99_; %T > MPC, the percentage of time that drug concentration remained above MPC. All PK/PD parameters were calculated as the mean values of multiple doses. Values are listed as mean ± SD.*

**Table 3:** Correlation of PK/PD parameters with selection of resistance.

PK/PD index, value	Fraction of tissue cages with resistant bacteria (mutant/total)	*P*
**AUC_24h_/MIC_99_(h)**		
<34.678	2/8	0.424
34.68–148.65	10/12	0.008
>148.65	1/8	0.667
**AUC_24h_/MPC(h)**		
<4.33	2/8	0.424
4.33–18.58	10/12	0.008
>18.58	1/8	0.667
**C_max_/MIC_99_**		
<1.09	0/4	NA
1.09–8.42	12/16	0.014
>8.42	1/8	0.667
**C_max_/MPC**		
<0.14	0/4	NA
0.14–1.05	12/16	0.014
>1.05	1/8	0.67
**T > MIC_99_**		
<17.15	2/8	0.424
>17.15	11/20	0.067
**T > MPC**		
<29.63	12/20	0.047
>29.63	1/8	0.667


*All PK/PD parameters were determined using total drug concentrations from the tissue cage fluid. A total of 28 tissue cages were analyzed. *P*-values were calculated using Fisher’s exact test, with a two infected but untreated piglets (four tissue cages) used as a control. NA, not applicable.*

Statistically significant correlations with selection of resistance for other PK/PD indices are also listed in **Table 3**. Mutants were selected by enrichment when the C_max_/MIC_99_ values were between 1.09 and 8.42 or C_max_/MPC values were between 0.14 and 1.05. Resistant bacteria were recovered from 12 of 20 TCs when the administration time of danofloxacin concentration was above the MPC for <29.63% of the dosing interval.

### Characterization of the Contribution of Efflux and Gene Mutations in QRDRs

Mutants selected from danofloxacin dosages of 0.6, 0.8, and 3.5 mg/kg tended to be non-topoisomerase mutants that exhibited increased efflux. This was confirmed by adding an efflux inhibitor (reserpine), which could decrease the MIC for danofloxacin and enrofloxacin (**Table 4**). Mutants obtained from 1.25 and 2.5 mg/kg dosages were less affected by reserpine (**Table 4**). Mutations in the QRDR target genes are listed in **Table 4**. No mutant genes were observed in *gyrB* and *parE*. All mutants had a (Lys-53→Glu) substitution in *parC*. When the dose was 0.6 and 0.8 mg/kg, no substitution was founded in *gyrA*. When the dose was 1.25 and 2.5 mg/kg, the mutants had a (Ser-83→Tyr) substitution in *gyrA*. When the dose was 3.5 mg/kg, the mutants had a (Ser-83→Phe) substitution in *gyrA*.

**Table 4:** Quinolone susceptibility and identification of resistant mutants associated with different dosages of danofloxacin.

	MICs (μg/mL)				Mutations	
Dosages (mg/kg)	Danofloxacin	Danofloxacin + reserpine	Enrofloxacin	Enrofloxacin + reserpine	*gyrA*	*parC*
0	0.06	0.03	0.125	0.03	–	–
0.6 (*n* = 2)	0.25	0.125	0.5	0.125	–	K53E
0.8 (*n* = 3)	0.25	0.125	0.5	0.125	–	K53E
1.25 (*n* = 4)	1	1	2	1	S83Y	K53E
2.5 (*n* = 3)	1	1	2	1	S83Y	K53E
3.5 (*n* = 1)	0.5	0.5	1	0.25	S83F	K53E


*–, No mutant was found; *n*, the number of tissue cages with mutant strains; *gyrA*, *parC*, the target genes of mutations in QRDRs. Reserpine concentration was at 20 μg/mL.*

## Discussion

Danofloxacin is a synthetic fluoroquinolone that was developed solely for veterinary therapeutic purposes and shows a wide spectrum of bactericidal activity that includes Gram-negative and some Gram-positive bacteria, mycoplasma, and intracellular pathogens such as Brucella and Chlamydia species (Sappal et al., 1996; Sunderland et al., 2003; Rowan et al., 2004). However, with the abundant application of antibiotics, antibacterial resistance has emerged as a serious public health problem in both humans and animals. One of the main reasons for this phenomenon is the inappropriate dosage regimens (dose, dosage interval, duration of treatment, routes, and conditions of administration; Toutain et al., 2002). Even the commonly accepted treatment strategy of killing susceptible pathogens contributes to the problem by stimulating selective amplification of resistant mutants during treatment (Stratton, 2003). Therefore, rational antibiotic dosing regimens should be optimized, not only to eradicate the culpable pathogens but they also have an important role in inhibiting the emergence and proliferation of antibiotic-resistant strains (Toutain et al., 2002). Therefore, we considered an exploration of the relationship between the MIC- and MPC-related PK/PD parameters and emergence of resistant mutants as being important in elucidating this phenomenon.

In the present study, both the susceptibility and resistant frequency of *A. pleuropneumoniae* increased when the concentration of danofloxacin exceeded MIC_99_ and below MPC. Compared with the changes in susceptibility, the resistant frequency of mutants increased dramatically when the concentration partially or completely decreased between the MIC_99_ and MPC (**Figures 3A2–A6**) in the present study. This phenomenon was also observed by other researchers (Cui et al., 2006; Zhu et al., 2012; Zhang et al., 2014a; Xiong et al., 2016). We postulate two reasons to explain this phenomenon. One reason may be the amplification of pre-existing resistant bacteria (Blondeau, 2009). When drug concentrations were located between MIC_99_ and MPC, the total population size reduced and then gradually re-constituted after several administrations of danofloxacin. The resistant frequency of mutants significantly increased but the number of mutants changed only slightly. These data indicated that a frequency increase may result from preferential killing of susceptible bacteria. Amplification of mutants was observed after several treatments (**Figure 2**). Another reason that could explain the increase in resistance frequency may due to gene mutations that arise in bacteria (Zhang et al., 2014a). After several applications of treatment, the sequence of nucleotides may change and a new mutant can be generated (Cui et al., 2006; Zhu et al., 2012). Consequently, the total population of bacteria was almost equal to the mutant population.

To assess the clinical effects and their potential in the prevention of antibiotic resistance development, antimicrobial PK/PD parameters have been used (Leroy et al., 2012). For fluoroquinolones, AUC_24h_/MIC can be applied commonly to predict favorable outcomes when susceptible populations are considered (Preston et al., 1998). And for MPC-related PK/PD indices, AUC_24h_/MPC is an appropriate parameter because MPC is the MIC of the least susceptible single-step mutant (Zhao and Drlica, 2001). In the present study, we considered keeping the value of AUC_24h_/MPC > 18.58 h as being a straightforward way to restrict the acquisition of resistance. The results fitted well with the conclusions of other researchers (Cui et al., 2006; Xiong et al., 2016). In an *in vivo* study, *Staphylococcus aureus* was treated with levofloxacin and AUC_24h_/MPC was also proposed. In their study, AUC_24h_/MPC > 25 h correlated with restricted growth of resistant mutant subpopulations (Cui et al., 2006). Another researcher studied the relationship between vancomycin and methicillin-resistant *S. aureus* (MRSA) *in vivo*. This group considered that resistant mutants were not enriched at a value of AUC_24h_/MPC > 15 h (Zhu et al., 2012).

Other PK/PD parameters such as C_max_/MIC_99_, C_max_/MPC, and T > MPC also exhibited a statistically significant correlation with resistance frequency. However, it is still not possible to accurately confirm the concentrations required to generate resistance in previously susceptible strains. For example, in an *in vitro* model, concentrations of antibiotics at the center between MIC_99_ and MPC were favorable in selecting a double mutant (Preston et al., 1998). In the present TC infection model, the concentration of danofloxacin required below MPC for 70.38% of the time to enrich mutants when those concentrations fluctuated above and below the MPC. However, the enrichment of mutants was observed when the concentration fluctuated above and below the MIC_99_ for only 17.15% of the interval time. One reason, which may explain this difference derives from more abundant pre-existing resistant mutant subpopulations being able to survive and expand near MIC99 (Zhou et al., 2000), while the mutants were killed when the drug concentration was near the MPC.

In Gram-negative bacteria, fluoroquinolone resistance occurs mainly by interplay of three mechanisms. This is realized by stepwise accumulation of mutations in the QRDRs of DNA gyrase and topoisomerase IV, active efflux of fluoroquinolones, and the presence of plasmid-borne resistance genes (qnr) protecting the target topoisomerase (Chu et al., 2005). In our experiment, the mutants had a (Ser-83→Tyr) or (Ser-83→Phe) substitution in *gyrA* and a (Lys-53→Glu) in *parC*. In a previous study (Wang et al., 2010), more mutant genes were found. They characterized the enrofloxacin-resistant *A. pleuropneumoniae* isolates and found seven different substitutions in GyrA (G75S, S83Y, S83F, S83V, D87Y, D87N, and D87H), four different substitutions in ParC (G83C, S85R, S85Y, and E89K), and five different substitutions in ParE (P440S, S459F, E461D, E461K, and D479E).

Although we successfully established a piglet TC infection model to evaluate the relationship between MIC- and MPC-based PK/PD parameters and the emergence of resistant mutants, there are some limitations to our study. First, because of the limited number of piglets, the sample size of resistant mutants is not enough to generalize. Larger datasets should be considered in future research. Second, although a TC infection model was suitable for exploring the relationship between PK/PD indices and antibacterial effects, there are still obvious differences between TCF and clinically infected organs in animals. Therefore, for *A. pleuropneumoniae*, a lung infection model may be preferable for the study of PD and PK information in future studies.

## Conclusion

We successfully established a piglet TC infection model and investigated the changes in susceptibility and mutant frequencies of *A. pleuropneumoniae* after different dosages of danofloxacin. After analyzing the relationship between MIC- and MPC-based PK/PD parameters and the emergence of resistant mutants, we suggest that danofloxacin concentrations should be maintained above the MPC or AUC_24h_/MPC > 18.58 h, which could maintain effective antibacterial activity and minimize the emergence of resistant *A. pleuropneumoniae*.

## Author Contributions

LZ and HD contributed to the methodology, software, validation, formal analysis, data curation, writing (original draft preparation), writing (review and editing), visualization, and the project administration. LZ, ZH, ZK, and LY contributed to the investigation. LZ, QC, XS, and HD contributed to the resources. XG and HD contributed to the supervision. HD contributed to the funding acquisition.

## Conflict of Interest Statement

The authors declare that the research was conducted in the absence of any commercial or financial relationships that could be construed as a potential conflict of interest.
